# Medical Fees and the Public

**Published:** 1909-10-30

**Authors:** 


### MEDICAL FEES AND THE PUBLIC.

*To the Editor of* The Hospital.

Dear Sir,—I was somewhat surpised to learn from your article upon the above subject, that the "average general practitioner's" fees are 'higher than they should be.'" Twelve years ago I started practice in a London suburb at 2s. 6d. for visit, and 1s. 6d. for advice and medicine in the surgery. Now I have new men surrounding me, who charge 4d., 6d., and 8d. in the surgery, and 5s. for midwifery.

To my mind the fees given to the general practitioner in this country are absolutely beneath contempt, and are little better than 'tips.' To keep one's self up to date requires considerable expense for books, instruments, etc., and, so far as I can gather, the public now expect everything for nothing, nor can this be wondered at in the face of the notorious Notification of Births Acts, which requires us to notify births, under a penalty of fine or imprisonment, and without any fee, for the sole benefit of the pocket of the district registrars, who have the right to inspect the book kept by the medical officer of health, wherein the notifications are recorded.

Yours truly,
"What Next."

[We referred more particularly to higher scales of fees—consulting fees—and definitely excluded from our expression of opinion such charges as our correspondent mentions. —Ed., The Hospital.]

